# Amoxicillin and Clarithromycin Mucoadhesive Delivery System for *Helicobacter pylori* Infection in a Mouse Model: Characterization, Pharmacokinetics, and Efficacy

**DOI:** 10.3390/pharmaceutics13020153

**Published:** 2021-01-24

**Authors:** Isabel Villegas, María Ángeles Rosillo, Catalina Alarcón-de-la-Lastra, Victoria Vázquez-Román, Maria Llorente, Susana Sánchez, Ana Gloria Gil, Pilar Alcalde, Esther González, Elisabet Rosell, Carles Nieto, Francisco Fernandez-Campos

**Affiliations:** 1 Department of Pharmacology, Faculty of Pharmacy, University of Seville, Profesor García González Street 2, 41012 Seville, Spain; villegas@us.es (I.V.); rosillo@us.es (M.Á.R.); calarcon@us.es (C.A.-d.-l.-L.); 2 Department of Normal and Pathological Cytology and Histology, Faculty of Medicine, University of Seville, Avda. Dr. Fedriani, s/n, 41009 Sevilla, Spain; mvazquez2@us.es; 3 Bionanoplus. Pol. Mocholí Plaza Cein Nº5, nave B14, 31110 Noáin, Spain; mllorente@bionanoplus.com (M.L.); ssanchez@bionanoplus.com (S.S.); 4 Toxicology Laboratory, DDUNAV Navarra University, Irunlarrea 1, 31008 Pamplona, Spain; agil@unav.es; 5 Reig Jofre Laboratories, Avda dels Flors s/n, 08970 Sant Joan Despi, Spain; palcalde@reigjofre.com (P.A.); erosell@reigjofre.com (E.R.); cnieto@reigjofre.com (C.N.); 6 Reig Jofre Laboratories, Rio Jarama 111, 45007 Toledo, Spain; gonzae00@reigjofre.com

**Keywords:** *Helicobacter pylori*, mucoadhesive, clarithromycin, amoxicillin, mice, pharmacokinetics, Mucolast^®^

## Abstract

*Helicobacter pylori* is the main pathogen responsible for gastric ulcers and a predisposing factor of stomach cancer. Although current treatment is usually successful, it requires high doses and frequent administration. An innovative mucoadhesive system (Mucolast^®^) loaded with amoxicillin and clarithromycin is proposed to improve the efficacy of treatment against *H. pylori*. The drug product was optimized based on its viscoelastic properties to obtain long-term stability of the vehicle. The drug release mechanisms were different for both antibiotics based on their solubilization status. A systemic and stomach pharmacokinetic profile was obtained after three different doses were administered to mice, obtaining similar systemic exposure levels but an increase in drug concentration in the stomach. The efficacy results in mice infected with *H. pylori* also demonstrated the superiority of the antibiotics when administered in Mucolast^®^, as shown by the bacterial count in stomach tissue and under histopathological and biochemical evaluation. The proposed treatment was efficacious and safe and is presented as a realistic alternative to current treatment options to improve patient compliance and to reduce bacterial resistance.

## 1. Introduction

*Helicobacter pylori* is a Gram-negative bacterium that is present in stomach mucosa in approximately 50% of the population [1] (some authors’ estimations increase this value to up to 80% of the population) [2]. In only 10% of cases does the colonization progress to gastric ulcer, and 1% of these could develop into gastric cancer. The high colonization ratios without an associated pathology support the hypothesis that *H. pylori* could be a commensal bacterium and that the progression into ulcers is associated with virulence factors of some strains [1] such as CagA positive (cytotoxin-associated gene A), VacA (vacuolating cytotoxin), adhesines, and surface glycoproteins (BabA, OipA, and SabA) [2]. The bacteria are characterized by the presence of flagella, which allow rapid movement from the lumen to the mucosa, and by urease-positive metabolism, which confers acid resistance in the stomach environment.

The primary clinical manifestation of *H. pylori* infection is acute gastritis. When it persists, chronic gastritis and environmental factors (diet, smoking and alcohol habits, anti-inflammatory medications, etc.) together with host-related factors (gene polymorphisms, immune response, etc.) lead to the development of gastric ulcers, pancreatitis, atrophy, and cancer [2].

Diagnosis of *H. pylori* infection usually involves the use of a gastric endoscopy (presence of mucosal inflammation and ulceration) and biopsy to perform immunohistochemical analysis to detect bacillus. As an alternative, a urease test of the breath is performed because it is a noninvasive and rapid screening tool that involves the administration of radio-labelled urea. Hydrolysis of orally ingested urea by *H. pylori* urease activity gives ^13^C-CO_2_, which diffuses blood, is released by the lungs, and is detected in exhaled air [3]. Once diagnosed, eradication treatment with antibiotics and/or proton pump inhibitors (PPI) is employed, which usually involves high drug doses and/or long periods. Treatments have been extensively discussed and reviewed in recent years because no treatment has claimed to eradicate *H. pylori* in 100% of individuals. Successful therapy is considered when a response has a higher than 90% eradication rate [4].

Dual therapy with PPI and amoxicillin (Amox) is currently less commonly used because it is believed that single antibiotics are related to a high rate of bacterial resistance. Triple therapy using PPI and a combination of two antibiotics has become the standard treatment, with Amox and clarithromycin (Clar), or metronidazole and Clar for 14 days being the most frequent administration scheme [5]. Other antibiotics are also employed, such as quinolones, tetracycline, and rifabutin [6]. The selection of Clar or metronidazole is based on a previous macrolide exposition because there is an increased amount of resistance for this antibiotic [4]. Several attempts have been made to reduce treatment duration to 7 or 10 days to improve patient compliance [6], but in both of those regimens, the eradication rate was lower compared to that for 14 days [5]. In certain cases, quadruple therapy is employed by adding bismuth salt to the PPI and two antibiotics. Bismuth interferes with the metabolism, reduces the adherence of bacteria to the gastrointestinal mucosa [7], and seems to increase the effectiveness of triple therapy, although due to the large heterogenicity of the studies, it is difficult to confirm and to compare treatments and durations [5,6].

Mucoadhesive formulations could have advantages compared to standard tablets for gastric ulcers caused by *H. pylori*. The increase in residence time in the gastric mucosa and the intimate contact of the vehicle with the site of action could increase the effectiveness of the treatment. Several authors have developed mucoadhesive micro and nanoparticles made of different polymers (chitosan [8,9], gliadin [10], and acrylic derivates [11]) to deliver a variety of compounds such as curcumin [12], other natural products [13,14], and different antibiotics [9,15,16]. Although these systems are promising nanoparticles characterized by a low loading capacity [17], it is difficult to encapsulate two or even three compounds in the same nanoparticle, especially when they share different physicochemical properties. Then, considering the high antibiotics doses and different antibiotic combinations required in *H. pylori* eradication, other approximations are required. A mucoadhesive anhydrous paste (Mucolast^®^ [18]) composed of carboxymethyl cellulose (CMC) and alginate has recently been developed and is claimed to have an improved therapeutic effect on gastrointestinal diseases. These polymers are not hydrated in glycerol, but upon contact with the water content of the gastric environment, they swell and adhere to the mucosa layer. While water is incorporated into the formulation, calcium sulfate salt, which is not soluble in the polyol liquid, dissociates and calcium is released in situ and cross-links with alginate. This increases the mucoadhesive force of the CMC and retains the antibiotics in the matrix, protecting them from the acidic gastric fluids. The system allows the loading of both Amox and Clar, protecting the former against degradation and adhering strongly to the mucosa, where bacterial infection takes place.

In this research, we characterized the formulation and studied the pharmacokinetic profile of Amox and Clar in the plasma and stomach tissue of antibiotics at different doses and their efficacy in a gastric ulceration mouse model caused by *H. pylori*. It is hypothesized that this system will maintain the systemic exposure of antibiotics and will increase their effectiveness due to the topical effect in gastric mucosa caused by direct contact of the drugs with bacteria.

## 2. Materials and Methods

### 2.1. Materials

Sodium alginate (Carlo Erba, Barcelona, Spain), sodium carboxy methyl cellulose (CMC) (Fagron, Barcelona, Spain), calcium sulfate (Panreac Química S.L.U., Madrid, Spain), glycerol (Barcelonesa Group, Barcelona, Spain), xanthan gum (Guinama, Valencia, Spain), and glycerol monostearate (Fagron, Barcelona, Spain) were used to produce the Mucolast^®^ vehicle, according to patent WO/2017/162822 “Compositions for mucosal adhesion and uses thereof” [18]. Amoxicillin trihydrate (Deretil S.A. Almeria, Spain) and clarithromycin (IND-SWIFT Laboratories Limited, Mohali, India) were mixed with the vehicle before administration.

### 2.2. Methods

#### 2.2.1. Drug Product and Characterization

Glycerol (q.s. 100%) was heated at 70 °C in a stainless-steel reactor equipped with water-jacked circulation. Glycerol monostearate (1%) was added; then, melted CMC (6%), alginate (4%), and calcium sulphate (1.25%) were subsequently added and homogenized for 40 min at 40 rpm with an anchor stirrer and 1500 rpm with a homogenizer stirrer. Lastly, xanthan gum (2–3%) was introduced and homogenized with the same stirring conditions. The swelling conditions of xanthan gum were determining factors that affected the stability of the final product; thus, a concentration range from 2% to 3% and hydration temperatures of 70 and 75 °C for 2 or 2.5 h were assayed. The obtained formulations were characterized in terms of rheology profile, mucoadhesion, release, and floatability.

Rheology characterization was evaluated by measuring viscosity and linear viscoelasticity properties using a DHR-1 rheometer (TA-Instruments, Barcelona, Spain) and parallel plate–plate geometry (PP25) with a gap of 2.0 mm and a Peltier plate set at 25 °C. Strain sweep measurements were performed with a strain range of 1–100% and frequency sweeps at a constant strain. Storage (G′), the slope of G′, and the delta tangent (δ) were obtained.

To determine mucoadhesion, a simulated mucosa with agar–agar and mucine was employed. Briefly, 17 g of agar–agar (Guinama, Barcelona, Spain) was mixed with 700 mL of water in a beaker. The mixture was stirred and heated to 95 °C for 15 min. After cooling at 70 °C, 2.5 g of type III pig mucin (Sigma, Barcelona, Spain) was added and the final mixture was homogenized. Finally, before reaching room temperature, 30 mL of the mixture was added to beakers with a 6 cm diameter and left to solidify at room temperature. Once the simulated mucosa was produced, 100 mg of Mucolast^®^ formulations was deposited on it and left to rest for 2 min. Then, the formulations were covered with 60 mL of simulated gastric fluid (2 g sodium chloride, 3.2 g purified pepsin, and 7 mL hydrochloric acid in 1 L of purified water, final pH = 1.2) and an orbital agitation at 150 rpm at 37 °C was applied. Mucoadhesion differences were obtained based on the time required to detach each formulation from the simulated gastric mucosa (indirect measurement of mucoadhesion as resistance to agitation, measured by the naked eye).

Floatability was determined by immersion of the formulations (100 mg) in 60 mL of the simulated gastric fluid in a crystal bake.

Finally, a dissolution test was performed to study the drug release profile of both antibiotics. For that purpose, 10 g of Mucolast^®^ loaded with Amox and Clar at three different doses was deposed in a dissolution apparatus of USP (United State Pharmacopoeia) class II (*n* = 3 for each dose and condition). The experimental conditions were based on USP monographs [19,20] adapted to the studied formulations. The doses were the same for the following pharmacokinetic study (89.4 and 44.7 mg/kg/day, 44.7 and 22.35 mg/kg/day, and 8.94 and 4.47 mg/kg/day for Amox and Clar, respectively). The dissolution vessels were filled with 450 mL of water or acetate buffer at pH 5 for Amox and Clar, respectively, to maintain sink conditions at 37 °C and under slight stirring at 50 rpm. Five milliliter samples were taken at regular time intervals (0.5, 1, 2, 3, 4, 5, and 6 h) and replenished with the same volume of dissolution medium at 37 °C. The samples were immediately analyzed according to the analytical method described in each monograph [19,20] and quantified with a calibration line with five standards between 500 to 0.5 µg/mL for Amox and between 250 to 0.25 µg/mL for Clar. Kinetic modelling of the dissolution data was studied with DD-solver Excel Add-in [21] using a nonlinear approach. The Akaike Information Criterion (AIC) was used to select the model that best explained the experimental data. Model parameter precision, expressed as CV% (standard deviation/mean ∙100), was also taken into account. The lowest AIC value and the lowest CV% had the best fit.

R_t_/R_∞_ = 1 − e^−kt^  First-order (1)

R_t_/R_∞_ = kt^1/2^    Higuchi’s equation (2)

R_t_/R_∞_ = 1 − e^−(t/td)β^   Weibull’s equation (3)

R_t_/R_∞_ = kt^n^    Korsmeyer-Peppas’s equation (4)

#### 2.2.2. PK Study

##### Animals and Treatments

C57BL/6 mice (25 g body weight, 9 weeks old) were used for the pharmacokinetic (PK) study. The animals were housed in standardized conditions, with a 12 h light/dark cycle (22 ± 2 °C; 50 ± 20% HR) with water and food ad libitum. Fifteen hours before the experiment, the food was retired. The study protocol (CEEA-009-15, approval date 30 September 2015) was approved by the Ethical Committee of University of Navarra.

Table 1 shows the animal distribution in the different experimental groups. Each group was composed of 30 animals, divided into subgroups (*n* = 5) according to the sacrifice time. Each time corresponds to one pharmacokinetic time point curve at which the blood and stomach were obtained. For this, 0.3 mL of blood was collected from the retro-orbicular plexus in EDTA-K_2_ tubes and centrifugated at 1200 rpm for 15 min at 25 °C to obtain plasma. Stomach mucosa was obtained by grazing the surface with a glass slide. A total of three sample types (plasma, stomach mucosa, and stomach tissue) were stored at −80 °C for a maximum period of 1 week, and the drugs were extracted and quantified according to the method shown in the next section.

The antibiotics were prepared in Mucolast^®^ vehicle or PBS solution immediately before administration. An orogastric tube was used to administer 100 µL of each treatment. At specified time points, the animals were euthanized by cervical dislocation and samples were obtained as previously described.

The doses of the PK study were selected after allometric conversion of the human dose (Amox and Clar human doses were 2 g/day and 1 g/day, respectively, divided by allometric factor 0.08) [22]. The highest tested dose in the PK study corresponds to one quarter of the equivalent human dose (after allometric conversion).

##### Drug Analysis with High-Performance Liquid Chromatography–Coupled Mass spectroscopy (HPLC-MS/MS)

Amox and Clar in the three different tissue matrices were analyzed by high-performance liquid chromatography–coupled mass spectroscopy (HPLC-MS/MS) (Waters 2795 XE Separations Module and, Micromass Quattro Ultima triple-stage quadrupole MS, Barcelona, Spain). Validation was carried out according to the Bioanalytical Method Validation guidelines [23].

Amox was analyzed with negative electrospray ionization under the MRM (Multiple Reaction Monitoring) mode. The mobile phase of 0.1 mM of ammonium acetate with formic acid 0.072 mM/acetonitrile (75:25) at 0.2 mL/min was eluted through a Luna C18 3 µm (100 × 2.0 mm) chromatographic column and heated at 30 °C. The injection volume was set at 5 µL. The validation linearity ranged from 50 to 25,000 ng/mL for Amox plasma samples and from 100 to 200,000 ng/g for Amox in mucosa and stomach tissue.

Clar was analyzed with positive electrospray ionization under the MRM mode. The mobile phase of 0.5 mM of ammonium acetate with formic acid 0.36 mM/acetonitrile (55:45) at 0.3 mL/min was eluted through the same column at 0.3 mL/min. The injection volume was 5 µL. The validation linearity ranged from 25 to 2000 ng/mL for Clar plasma samples and from 50 to 100,000 ng/g for Clar in mucosa and stomach tissue.

Before the analysis, the samples were warmed and proteins were precipitated with acetonitrile in the case of plasma samples (sample volume 25 µL) and with methanol for both stomach samples (sample amount 15 mg). Additional appropriate dilutions were performed to obtain concentrations within the validation range. Ampicillin trihydrate (Sigma-Aldrich, Barcelona, Spain) was used as an internal standard spiking with 0.1 mL of a stock solution of 0.5 µg/mL in acetonitrile (Ultra Gradient HPLC Grade).

#### 2.2.3. PD Study

##### In Vitro Antibacterial Study of Amox and Clar

The in vitro antibacterial effect of Amox and Clar was determined based on the *H. pylori* SS1 strain by the microdilution method based on (and adjusted to this strain) the methodology described and validated by Ferreira da Silva et al. [14] and Goswami et al. [24]. The bacteria were kindly gifted by Dr. Liden from Gothenburg University (Sweden). The antibiotics stock solutions of 5 mg/mL in PBS for Amox and in methanol for Clar were subsequently diluted in PBS from 500 to 0.00095 µg/mL in 96-well plates. *H. pylori* was cultured in Brucella broth liquid medium supplemented with fetal bovine serum (5%) and an antibiotic mix (24 μL) containing amphotericin B (4 mg/mL), vancomycin (10 mg/mL), and trimethoprim (5 mg/mL) in order to avoid the growth of other microorganisms for 24 h at 37 °C under continuous stirring (1500 rpm) in a microaerophilic atmosphere (10% CO_2_, 85% N_2_, and 5% O_2_) (Campygen^®^, ThermoScientific-Walthem, Massachusetts, USA). The bacteria stock solution concentration was adjusted to 10^9^ CFU/mL (Colony Forming Units/mL). In total, 10 µL of inoculum was added to the 96-well plates and incubated in the same conditions (with a final bacteria concentration of 10^7^ CFU/well). Absorbance at 600 nm was determined with a spectrophotometer (Synergy HT spectrophotometer, Colmar Cedex, France) after 24, 48, and 72 h. Methanol and PBS blank samples were used as controls with the same dilution scheme as previously described. The first concentration value with no bacterial growth was selected.

### 2.3. Animals, Infection Model, and Treatments

C57BL/6 mice (female, 25 g body weight, 10 weeks old) were used for the efficacy study. Animals were housed in standardized conditions, with a 12 h light/dark cycle (22 ± 2 °C; 50 ± 20% HR) with water and food ad libitum. The study protocol (CEEA-US2015-33/2, approval date: 18 March 2016) was approved by the Ethical Committee of Sevilla University.

The animal infection model of *H. pylori* was adapted from Navabi et al. [25]. Briefly, a single dose of 300 µL of bacterial suspension at 10^9^ CFU/mL was administered to animals by an orogastric tube (FTP-20-38-50, Instech, Winsum, Netherlands). Infection was confirmed during the development of this method by counting the CFU in tissue two and three weeks after administration.

Two weeks after bacterial inoculation, 0.1 mL of each formulation was administrated daily (one dose per day) for five days. The animals were sacrificed two days after the last treatment administration, and their stomachs were taken for CFU counting and immunohistochemistry study. The treatments are described in Table 2.

The animals were sacrificed by cervical dislocation 48 h after the last antibiotic administration, and their stomachs were taken and cleaned. The organ was cut by its major curvature, dividing it into two equal parts longitudinally. One piece was used for histological/biochemical analysis, and the other section was homogenized in 1 mL of sterile PBS; further dilutions were performed from 10^−2^ to 10^−7^. In total, 0.1 mL of each dilution was placed in a selective medium, modified with BD Helicobacter (BD Biosciences—Franklin Lakes, NJ, USA), and incubated for 7 days in microaerophilic conditions (as described previously) at 37 °C.

Dose selection in the PD studies was based on the human dose after allometric conversion, as previously described in the PK studies. As it will be shown in the Results section, the PD 6 group did not show any antibacterial effect; thus, the lowest dose used in the PK study (Amox 8.94 mg/kg/day and Clar 4.47 mg/kg/day) was not tested because no effect was expected. To obtain a dose—response relationship, it was decided to test the group PD 4 for a high antibacterial effect. This dose corresponds to the equivalent human dose after allometric conversion.

### 2.4. Histology

The histopathological and immunohistochemical study was conducted on the portions of stomachs (and the adjacent exophages portion) dissected on the day of the animal’s sacrifice and fixed in 4% formol for 24 h. Afterward, the tissue was dehydrated through a series of washes with ethanol from 50° to 100° for later inclusion in paraffin. Cuts of 4 mm of the paraffin inclusions were made with a microtome (Microm Heidelberg^®^). Subsequently, the respective protocols were followed in order to carry out different staining types: hematoxylin/eosin staining (H-E) [26], Giemsa staining, PAS staining (Periodic Acid-Schiff), and Immunohistochemistry–Anti-*H. pylori* antibody using the anti-HP Ac antibody polyclonal in rabbit dilution 1/200 (Novus Biologicals, Littleton, CO, USA) and following the protocol described in the study published by Vazquez-Román et al. [27]. The revealing system used was Flex EnVision.

### 2.5. Biomarkers

The analysis of biochemical parameters was performed using the portions of stomachs preserved at −80 °C from the day of sacrifice. These stomachs were homogenized in buffer A (Hepes 1 M, EDTA 0.1 M, Ethyleneglycol-*bis*(β-aminoethyl)-N,N,Nʹ,Nʹ-tetraacetic Acid (EGTA) 0.01 M, KCl 1 M, dithiothreitol (DTT) 1 M, Sodium Fluoride (NaF) 0.5 M, Na_3_VO_4_ 0.5 M, Leupeptin 1 mg/mL, aprotinin 100 µg/mL, and Phenylmethanesulfonyl Fluoride (PMSF) 0.2 M). Subsequently, they were sonicated at 4 °C in three cycles of 10 s and centrifuged at 2500 rpm for 15 min at 4 °C. Different aliquots of the supernatants were preserved at −80 °C.

### 2.6. Enzyme-Linked Immunosorbent Assay

The concentrations of interleukin (IL)-1β and tumor necrosis factor alpha (TNF-α) in stomach homogenates were determined using appropriate commercial enzyme linked immunosorbent assay (ELISA) kits for the murine form of these two proinflammatory cytokines (Diaclone, Besançon, Cedex, France) as well as prostaglandin E2 (PGE2) levels (Cayman Chemical Company). The homogenates were centrifuged (4000× *g*, 10 min, 4 °C), and the supernatants were used for determination. All measurements were carried out in duplicate. The results were measured in picograms per milliliter at 450 nm using an ELISA microplate reader (BioTek, Bad Friedrichshall, Germany).

### 2.7. Western Blot Analysis

The protein concentration of the stomach homogenate was determined following Bradford’s colorimetric method [28]. Aliquots of the supernatants containing equal amounts of protein (50 mg) were separated on a 10% acrylamide gel by sodium dodecyl sulfate–polyacryamide gel electrophoresis. In the next step, the proteins were electrophoretically transferred onto a nitrocellulose membrane and incubated with the specific primary antibody rabbit anti-ciclooxygenase-2 (COX-2) (Cayman, Ann Arbor, MI, USA), at a dilution of 1:2500 overnight at 4 °C. After that, the membrane was washed three times for 15 min and incubated with a horseradish peroxidase (HRP)-labeled secondary antibody anti-rabbit (Cayman Chemical, Ann Arbor, MI, USA) at a dilution of 1:2500 containing blocking solution for 1–2 h at room temperature. To prove equal loading, the blot was analyzed for β-actin expression using an anti-β-actin antibody (Sigma Aldrich, St. Louis, MO, USA). Immunodetection was performed using an enhanced chemiluminescence light-detecting kit (Pierce, Rockford, IL, USA). Then, the immunosignals were captured using an LAS-3000 Imaging System from Fujifilm Image Reader (Mac™ and Windows), and the densitometric data were studied following normalization to the control (house-keeping gene). The signals were analyzed and quantified by Image Processing and Analysis in Java (Image J, Softonic, USA) and expressed as a percentage with respect to the control group.

## 3. Results

### 3.1. Drug Product and Administration

The Mucolast^®^ vehicle was successfully obtained according to the procedure described in Section 2.2. As a result, a smooth and homogeneous anhydrous emulsion was obtained.

The antibiotics were mixed with the vehicle, and the stability of both compounds was followed over time at 4 °C, 25 °C, and 40 °C. After 18 days of storage at these conditions, there were no quantifiable levels of Amox at 25 °C and 40 °C and there was a loss of 40% in the initial values when the product was stored at 4 °C. Thus, Amox is not considered stable with the vehicle, and extemporary administration should be considered. The antibiotics are easily stirred with a spatula for approximately one minute just before administration.

The results of the viscosity, G′, G′ slope, δ tangent, mucoadhesion, and floatability are presented in Table 3.

Previous experiments (data not shown) demonstrated that a xanthan gum hydration temperature below 70 °C caused sedimentation of the polymers and that temperatures over 75 °C led to excessive viscosity, which made the formulation difficult to handle and swallow. In all the obtained formulations, when stored at 4 °C and 40 °C for one month, viscosity increased dramatically, and the formulations were considered not stable at these temperatures. At 25 °C, there was a slight increase in viscosity that did not affect product performance.

The lack of floatability, observed in all formulations, is an important characteristic of the formulation because gastric ulcers are mainly located in the distal section of the stomach antrum [29]; thus, an increase in the contact time of the vehicle with the gastric mucosa is expected in this case.

Viscoelasticity studies described the microstructure of the delivery system and were able to predict the product stability over time. G′ is related to the internal formulation structure, since with a higher G′, a more stable formulation is expected. Lower G′ slope values are related to higher product stability. Tangent δ values quantified the balance between energy loss and storage. This value should be between 0.5 and 1.5, and within this range, a lower value indicates a higher structure and therefore more stability. A general linear model was developed to study the factors that influenced the rheological parameters. In any case, statistical differences were observed regarding the homogenization time. The G′ slope and regression coefficient were not affected by any of the studied factors (*p* > 0.05). Viscosity and δ were only affected by the percentage of xanthan gum (*p* < 0.05), and G′ was mainly affected by the percentage of xanthan gum (*p* < 0.01) and, to a lesser extent, by the hydration temperature (*p* < 0.1). With a higher percentage of xanthan gum (formulations 3 and 4), a greater structure (G′) is observed in the formulation but lower δ values are obtained, and thus, both formulations are expected not to be stable after long-term observations.

As a result, considering all previous described parameters, formulation 4 was selected as the final formulation.

**Figure 1:**
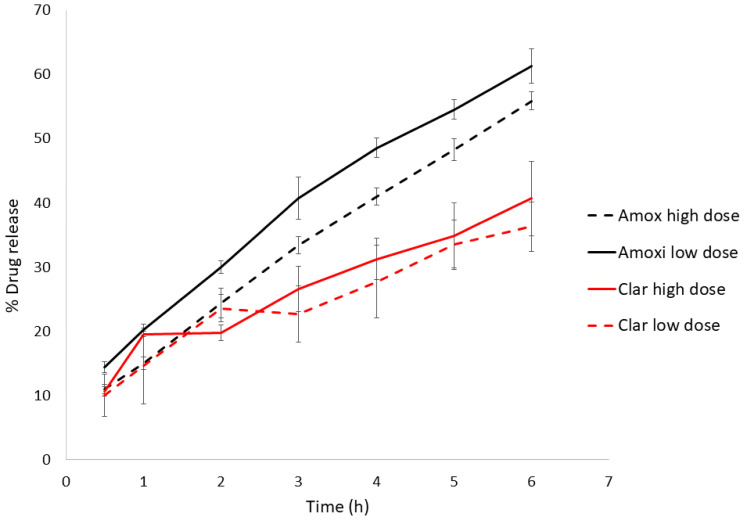
Highest and lowest dose dissolution profiles of Amox and Clar in Mucolast^®^. High dose: 89.4 mg/kg/day of Amox and 44.7 mg/kg/day of Clar. Low dose: 8.94 mg/kg/day of Amox and 4.47 mg/kg/day of Clar.

The dissolution test was performed on the highest and lowest antibiotics doses (Figure 1) to check the effect of drug concentration in the vehicle. Table 4 shows the model selection and parameters. Amox release from the Mucolast^®^ vehicle followed a Korsmeyer–Peppas (KP) equation at both doses with an exponent between 0.5 and 1, corresponding to an anomalous transport, which was probably due to a combined release mechanism. Amox is partially solubilized in glycerol; the higher the dose, the more drug is suspended. The Fickian diffusion of the solubilized faction (a function of drug gradient concentration) could be one of the release mechanisms of the drug, combined with the release of the suspended fraction. This fraction is probably coated by formulation polymers, making it less accessible to free water and delaying its release to a greater extant compared with the solubilized fraction, obtaining a combined release mechanism. This could also explain the lower release rate (the KP slope observed in Table 4) obtained in the higher dose compared with the lower dose (with statistically significant differences, *p* < 0.05). On the other hand, Clar at both concentrations followed a Higuchi equation (with no statistical differences, *p* > 0.05). This equation has been typically used to described the release of ointment drugs, in which the compound exceeds the solubilization in the matrix [30]. Clar was suspended in the vehicle, and its release followed a pseudo-steady state, making the diffusion coefficient constant.

### 3.2. PK Study

The selection of doses was based on human doses. Standard Amox and Clar human doses are 2 g/day and 1 g/day, respectively, divided in two doses every 12 h. Considering an average human weight of 70 kg, the resulting doses were 28.57 mg/kg/day for Amox and 14.29 mg/kg/day. Considering that bigger animals have a slower metabolic rate and that their metabolic process are slower than smaller animals, a conversion (dividing the human dose by allometric factor 0.08) [22] of the dose from a standard human dose to dose administrated on mice was carried out. Then, the equivalent standard human doses to mouse doses were 357.14 mg/kg/day for Amox and 178.57 mg/kg/day of Clar. The proposed mucoadhesive formulation was expected to have a better therapeutical effect compared to the standard non-mucoadhesive formulation; thus, the higher dose used in preclinical studies was one-quarter of the standard dose.

**Figure 2:**
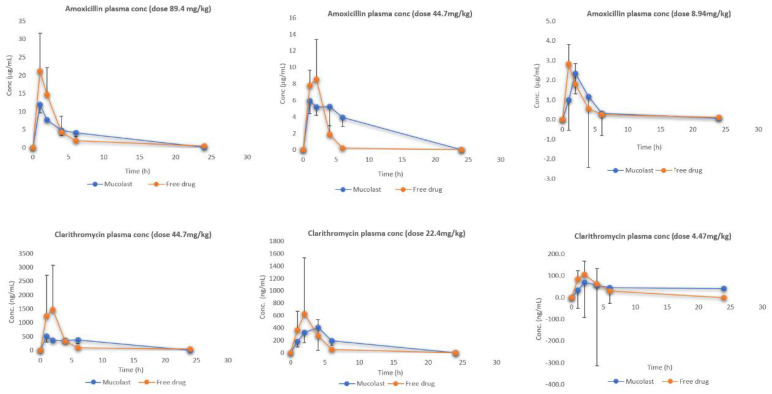
Amox and Clar plasma levels after the administration of antibiotics in Mucolast^®^ or PBS solution at three different doses.

Figure 2 shows the plasma levels of Amox and Clar at three dose levels. After the administration of free antibiotics, higher plasma levels (C_max_) were obtained compared with the antibiotics loaded in the Mucolast^®^ vehicle, as expected, because absorption in the formulation group was retarded due to mucoadhesion. Differences between formulations regarding C_max_ decreased as the dose was reduced. Few differences were observed regarding T_max_. The possible reason for this observation is that there was a portion of antibiotics that did not adhere to the stomach because the relationship between formulation volume/stomach surface and empty gastric mucosa caused this fraction to be absorbed in the duodenum. The fraction in contact with the stomach mucosa was absorbed later because of the adhesion of the polymers to the tissue. These facts caused a reduced C_max_ but not delayed T_max_.

When observing the area under the curve (AUC_all_), there are some differences between free and Mucolast^®^-loaded antibiotic plasma levels (Table 5). The AUC ratio between groups PK4 and PK1 was 98.9%, between groups PK5 and PK2 was 41.7%, and between groups PK6 and PK3 was 95.5% for Amox, and these values were 99.2%, 69.0%, and 62.0% for Clar, respectively. It was not expected to have different AUCs between free and loaded antibiotics, but as each time point came from different animals, there was a high interindividual variability associated with the experimental design.

**Figure 3:**
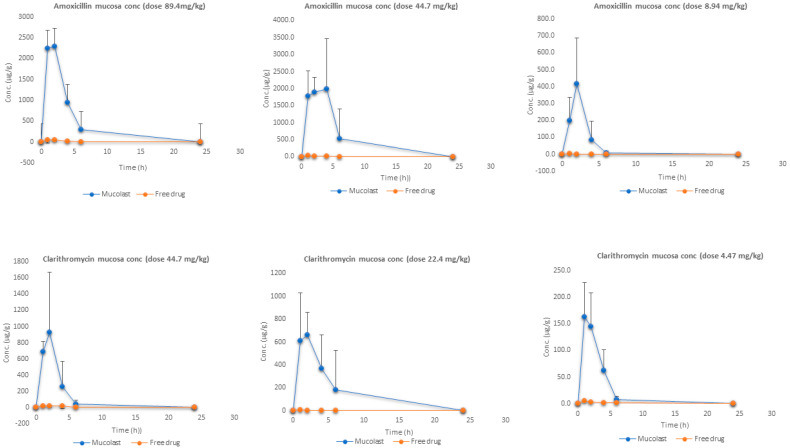
Amox and Clar mucosa levels after the administration of antibiotics in Mucolast^®^ or saline solution at three different doses.

Figure 3 shows the mucosa stomach levels of Amox and Clar at three doses. As expected, the antibiotic present in gastric mucosa after the treatment with Mucolast^®^ vehicle exhibited a high concentration compared with free antibiotics due to the mucoadhesive nature of the vehicle. The AUC_all_ ratio (Table 6) values between free and loaded Amox ranged from 0.6% to 2.9%, which represented a fold increase in drug exposure in the gastric mucosa between 30 to 170. The AUC_all_ ratios for Clar between free and loaded antibiotics were between 0.7% and 3.4%—approximately an exposure increase of 30 to 150 times. It is expected that a higher drug concentration in the mucosa for an extended time could lead to an increased bactericidal effect.

When a drug is administrated as a solution, the contact time between the mucosa and solution is very low and a topical absorption is not expected to happen; the drug present in this tissue is obtained from systemic circulation once the compounds are absorbed. On the other hand, the high mucosa exposure after mucoadhesive formulation came to a higher extent from the topical entrapment in the gastric environment and a small portion (the same as free solution) was due to systemic circulation.

**Figure 4:**
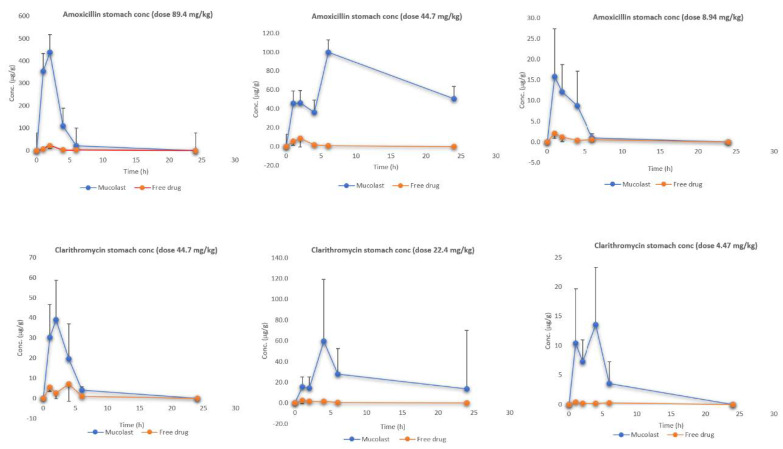
Amox and Clar stomach levels after the administration of antibiotics in Mucolast^®^ or saline solution at three different doses.

Figure 4 shows the stomach (under mucosa tissue) levels of Amox and Clar at three doses. Similar to the mucosa results, the antibiotic stomach values were higher after Mucolast^®^ administration compared with the free drugs. In this case, the AUC_all_-enhanced values (Table 7) were lower than those found in the mucosa and ranged approximately from 5 to 50. In all cases, the concentrations found in the stomach were lower compared with the mucosa. In the same way as previously described, the drug found in the stomach tissue when free antibiotics were administered came from systemic circulation, whereas when administered in mucoadhesive formulation, they came from both systemic circulation (in a small proportion) and from topical absorption. In this case, as the stomach tissue is deeper than the mucosa, the partitioning of the drug into it is smaller in comparison.

No previous literature was found describing the pharmacokinetic profile of both antibiotics in gastric tissue in mice.

### 3.3. PD Study

#### 3.3.1. In Vitro Antibacterial Study of Amox and Clar

An in vitro antibacterial study was performed on the *H. pylori* strain SS1 to evaluate its susceptibility to Amox and Clar. This strain expresses VacA and CagA virulence factors. The value of Amox susceptibility was set between 0.5 and 1 µg/mL, and that of Clar was set between 0.06 and 0.1 µg/mL. These values were constant over 24, 48, and 72 h. According to the European Committee on Antimicrobial Susceptibility Testing (EUCAST) [31], *H. pylori* is considered resistant to Amox if the MIC value is higher than 0.125 µg/mL and to Clar if the MIC is higher than 0.5 µg/mL. Thus, the strain SS1 used in the present study could be considered resistant to Amox but sensitive to Clar.

#### 3.3.2. In Vivo Study

Before animal treatment with antibiotics, the infection model was verified. Following the model described by Navabi et al. [25], 300 µL of *H. pylori* inoculum at 10^9^ CFU/mL was administered at time zero. The animals were sacrificed at 14 and 21 days after bacterial administration to evaluate the infection status and the bacterial recovery. The control animals were inoculated with the same volume of PBS solution. In addition, another control of the Mucolast^®^ paste was assayed to check that the vehicle did not affect the mucosal structure. After sacrifice, the stomachs were homogenized in 1 mL of sterile PBS, serial dilution was carried out, and 0.1 mL of each dilution was placed in a BD Helicobacter modified agar to obtain final concentrations ranging from dilutions of 10^−2^ to 10^−7^. The plates were incubated at 37 °C for 7 days in microaerophilic conditions, and CFUs were counted (Table 8). As no statistical differences (*p* < 0.05) were found between both time periods, a sacrifice at 14 days after inoculum was selected. Then, the final infection model involved the inoculation of 300 µL of *H. pylori* at 10^9^ CFU/mL and then leaving the animal for 14 days. At that point, the antibiotic treatment was administrated for 5 days and mice were sacrificed 48 h after the last treatment administration. The stomach was then divided longitudinally in two sections: one section was used to quantify the CFU/g, and the other section was used to perform histological and immunohistochemical evaluation.

**Figure 5:**
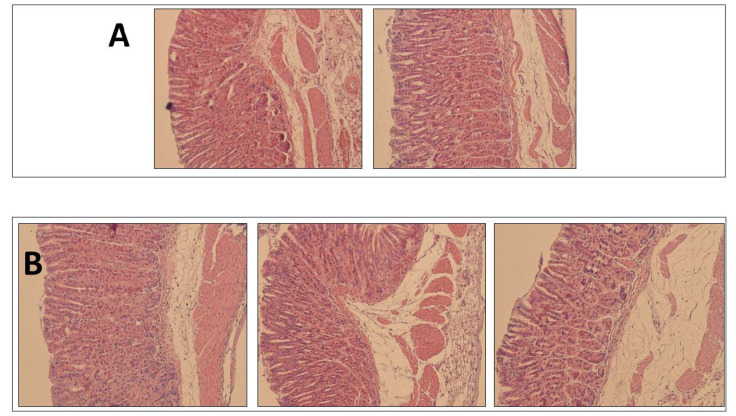
Histological examination of health gastric mucosa after PBS (**A**) of Mucolast^®^ (**B**) administration.

Figure 5 shows the histological examination of the gastric mucosa after inoculation of the PBS control and Mucolast^®^ control, and no damage signs were observed in both cases; thus, it could be concluded that the vehicle is compatible with the gastric mucosa.

**Figure 6:**
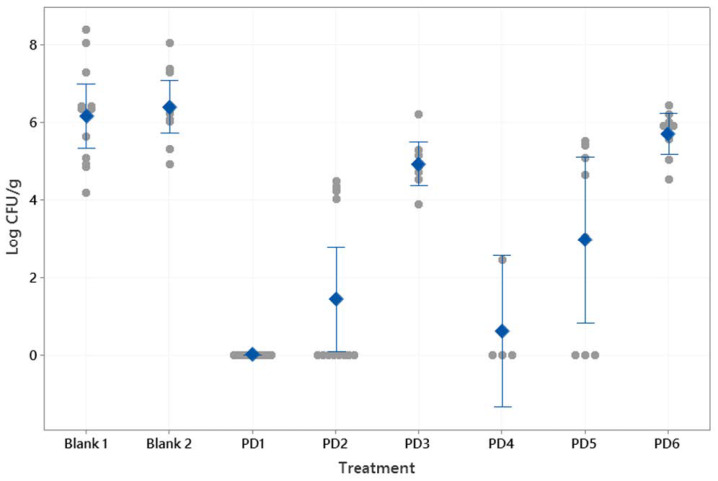
Bacterial count (CFU/g) found in mice stomachs after antibiotic treatments at different doses and controls (PBS and Mucolast^®^). Circles: individual CFU/g values. Diamonds: mean value. Interval bars: 95% confidence interval. Blank 1: Mucolast^®^. Blank 2: PBS. PD1: 89.4 mg/kg/day Amox and 44.7 mg/kg/day Clar in Mucolast^®^. PD2: Amox 44.7 mg/kg/day and Clar 22.35 mg/kg/day in Mucolast^®^. PD3: Amox 8.94 mg/kg/day and Clar 4.47 mg/kg/day in Mucolast^®^. PD4: Amox 357.5 mg/kg/day and Clar 178.8 mg/kg/day in PBS. PD5: Amox 89.4 mg/kg/day and Clar 44.7 mg/kg/day in PBS. PD6: Amox 44.7 mg/kg/day and Clar 22.35 mg/kg/day in PBS.

Figure 6 shows the CFU/g results after the treatments described in Table 2. The control samples (saline solution and Mucolast^®^ vehicle) obtained similar levels of CFU/g (p > 0.05). In general, as the antibiotic dose decreased, there was an increase in *Helicobacter* infection. At doses of 89.4 mg/kg/day of Amox and 44.7 mg/kg/day of Clar, the differences between drugs in saline solution (2.96 CFU/g) and in Mucolast^®^ (0 CFU/g) were high (*p* < 0.01), showing that administration of the antibiotics in the mucoadhesive vehicle improves their efficacy, which is probably due to the prolonged contact time with the stomach, leading to a topical effect in addition to the systemic effect. When the doses decreased to 44.7 and 22.35 mg/kg/day (Amox and Clar, respectively) in Mucolast^®^, the bacterial count increased to around 1.42 CFU/g; when this dose was administrated in saline solution, there was no inhibition (5.70 CFU/g). Doses equivalent to 8.94 and 4.47 mg/kg/day showed no inhibition of bacteria in either vehicle. The antibiotics in PBS can only eradicate the infection when administrated at an equivalent human dose of 357.5 mg/kg/day and 178.8 mg/kg/day for Amox and Clar, respectively. That dose was not tested in the Mucolast^®^ vehicle.

**Figure 7:**
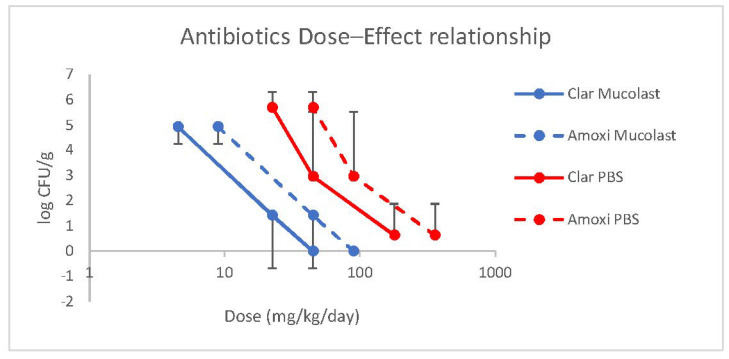
Amox and Clar dose–effect relationship of Mucolast^®^ and PBS.

A linear relationship between antibiotic doses loaded in Mucolast^®^ and efficacy (expressed as CFU/g) was found (Figure 7) with a regression equation of the log-transformed dose of y = 3.79–0.46 for the Clar dose (r^2^ = 0.99983) and y = 4.48–0.47 for the Amox dose (r^2^ = 0.99984). For antibiotics in saline solution, the linear regressions found were y = 5.28–0.405 for the Clar dose (r^2^ = 0.9453) and y = 5.98–0.406 for the Amox dose (r^2^ = 0.9457).

Current treatments of *H. pylori* aim to eradicate the infection. This is an objective that is difficult to achieve for several reasons. On the one hand, because the pathogen has multiple resistances, there are difficulties getting the therapeutic drug concentration into the mucosal layer, where the bacteria are protected due to systemic circulation, along with difficulties in patient adherence to treatment because a high antibiotic dose and a high number of administrations are usually required (leading to local and systemic adverse effects). The results shown in this study demonstrated that complete eradication is obtained with one-quarter of the required dose when drugs are loaded into the mucoadhesive vehicle compared with free antibiotics. The optimization of drug regimens with formulations specially designed for specific pathologies would improve patient compliance and clinical efficacy, with a lower adverse effect profile and a reduction of the likelihood of bacterial resistance.

##### Histological Evaluation

Several histological stains were performed on the gastric tissue and on part of the esophagus proximal to the stomach. The esophagus was included as a control because *H. pylori* could be retained in the keratin of the esophagus epithelium when the bacterium is inoculated by the oral–gastric canula.

**Figure 8:**
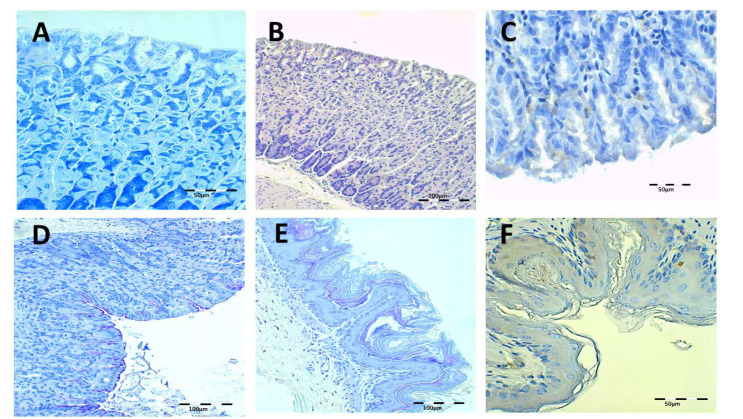
Healthy control animal microphotographs of the stomach (**A**–**D**) and esophagus (**E**,**F**), stained with Giemsa (**A**), hematoxylin/eosin staining (H–E) (**B**,**E**), immunohistochemistry (IHC) (**C**,**F**), and Periodic Acid-Schiff (PAS) (**D**).

Health control animal microphotographs are shown in Figure 8. H-E staining shows the normal histological structure of the stomach, while PAS images show the intact mucosa lamina covering the gastric foveolas. In both stainings, the mucosa and submucosa connective tissue showed no alterations. The Giemsa and immunohistochemistry (IHC) images do not identify *H. pylori* bacillus.

**Figure 9:**
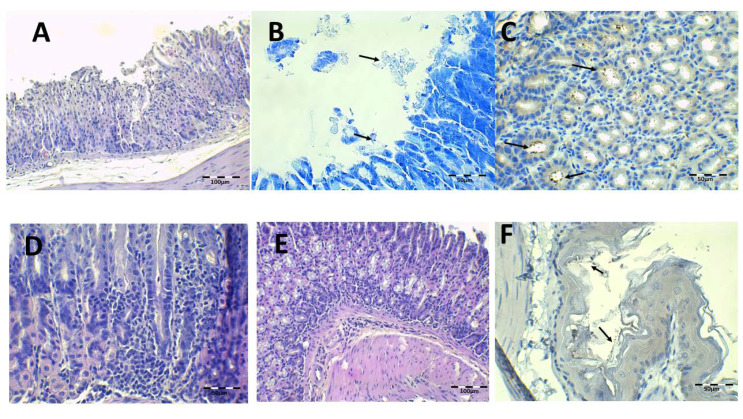
Control, PBS-infected animal microphotographs of the stomach (**A**–**E**) and esophagus (**F**) stained with H-E (**A**,**D**,**E**), Giemsa (**B**, arrows indicate *H. pylori* bacteria), and IHC (**C**,**F**, arrows indicate *H. pylori* bacteria).

The infected animals treated with saline solution and without antibiotics are shown in Figure 9. Under H-E staining, there was damage to the gastric mucosa with the loss of individual cells with a desquamative appearance or even loss of complete mucosa pieces (Figure 9A). The atrophy and epithelial destruction were related to the virulence factors CagA and VacA present in strain SS1 used in this study. Leucocyte infiltration in the lamina propria of mucosa and submucosa can also be observed (Figure 9D,E). Bacillus are identified by IHC in gastric foveolas and in the gastric surface with Giemsa (Figure 9B,C,F). They are also observed in the esophagus under both staining methods.

**Figure 10:**
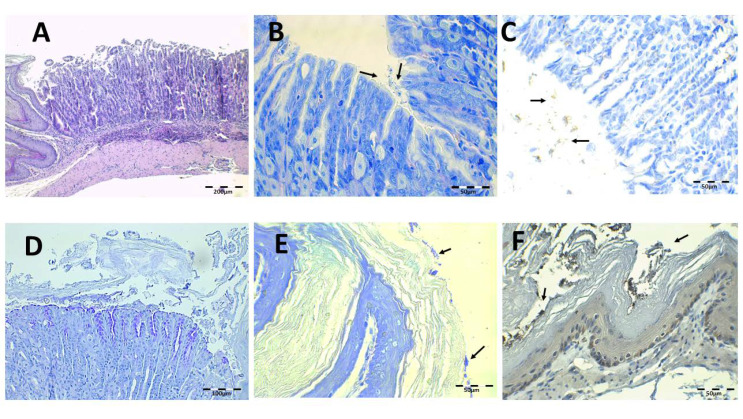
Control Mucolast^®^-infected animal microphotographs of the stomach (**A**–**D**) and esophagus (**E**,**F**) stained with H-E (**A**), Giemsa (**B**, arrows indicate *H. pylori* bacteria), IHC (**C**,**F**, arrows indicate *H. pylori* bacteria), and PAS (**D**).

The results of the infected animals administered with the Mucolast^®^ vehicle without antibiotics are shown in Figure 10. There are similar findings to the saline solution group. The PAS images showed the presence of the vehicle adherent to the mucosa surface (Figure 10D). It seems that the paste retained the desquamative cells of the epithelium caused by the bacteria.

**Figure 11:**
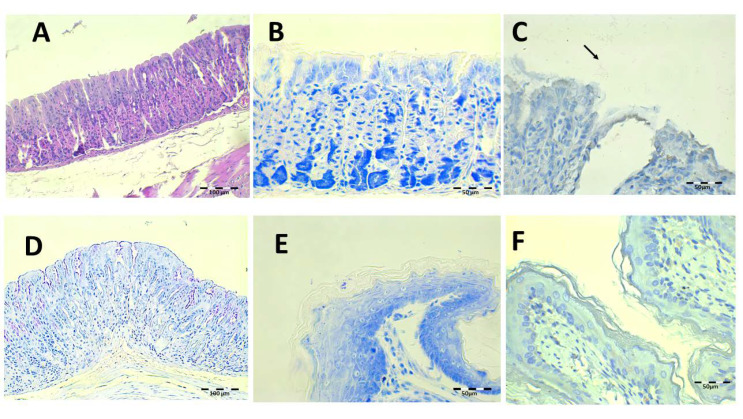
Infected animals treated with 89.4 mg/kg/day of Amox and 44.7 mg/kg/day of Clar in PBS microphotographs of the stomach (**A**–**D**) and esophagus (**E**,**F**), stained with H-E (**A**), Giemsa (**B**,**E**), IHC (**C**,**F**, arrows indicate *H. pylori* bacteria), and PAS (**D**).

The histology results after treatment with the saline solution with 89.4 mg/kg/day of Amox and 44.7 mg/kg/day of Clar are shown in Figure 11. Epithelial erosion and desquamation are hardly present under H-E staining (Figure 11A). There are no signs of leucocyte infiltration in the connective tissue. PAS showed continuous lamina over gastric foveolas expected over the erosive areas (Figure 11D). No bacillus fragments were identified by Giemsa or IHC, except for little bacteria groups captured in the keratin of the esophagus.

**Figure 12:**
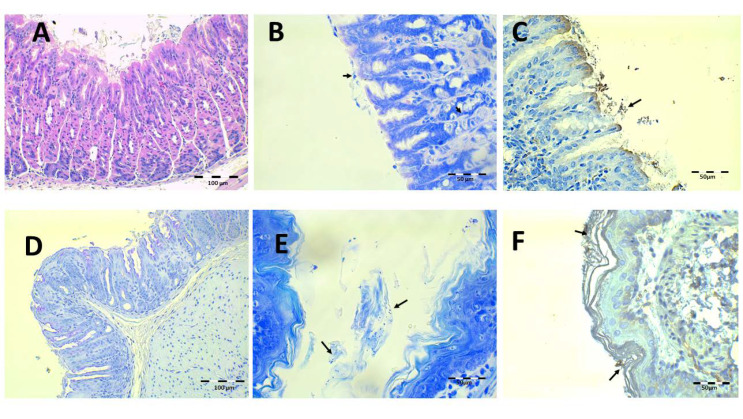
Infected animals treated with 89.4 mg/kg/day of Amox and 44.7 mg/kg/day of Clar in Mucolast^®^ microphotographs of the stomach (**A**–**D**) and esophagus (**E**,**F**), stained with H-E (**A**), Giemsa (**B**,**E**, arrows indicate *H. pylori* bacteria), IHC (**C**,**F**, arrows indicate *H. pylori* bacteria), and PAS (**D**).

The results after treatment with Mucolast^®^ at 89.4 mg/kg/day of Amox and 44.7 mg/kg/day of Clar are shown in Figure 12. Some paste can be observed to rest over the gastric epithelium. In general, the histologic structure under H-E (Figure 12A) is normal and there is no discontinuity under PAS staining (Figure 12D). In the Giemsa (Figure 12B,E) and IHC (Figure 12C,F) samples, little bacillus groups could be seen within the paste stuck in the stomach and esophagus. It seems that the vehicle retained bacterial colonies but that it is difficult to state if these colonies are active or inactive; however, probably, considering the results of CFU/g previously described, the bacteria were dead.

**Figure 13:**
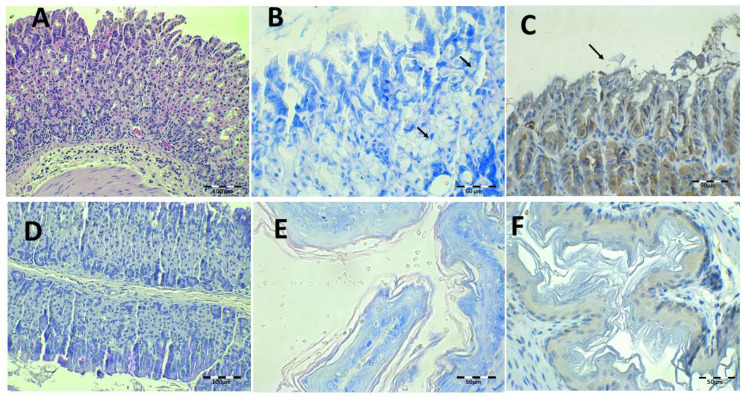
Infected animals treated with 44.7 mg/kg/day of Amox and 22.35 mg/kg/day of Clar in PBS microphotographs of the stomach (**A**–**D**) and esophagus (**E**,**F**), stained with H-E (**A**), Giemsa (**B**,**E**, arrows indicate *H. pylori* bacteria), IHC (**C**,**F**, arrows indicate *H. pylori* bacteria), and PAS (**D**).

The findings obtained after saline solution treatment at 44.7 and 22.35 mg/kg/day of Amox and Clar, respectively, are shown in Figure 13. There are small mucosa alterations and no epithelium alterations or discontinuity of mucosa laminae (Figure 13A,D), but there are inflammatory infiltrations in the mucosa and submucosa tissue. There is the presence of some isolated bacillus under Giemsa and IHC (Figure 13B,C), as expected based on the previous results obtained after counting the CFU in the stomach tissue.

**Figure 14:**
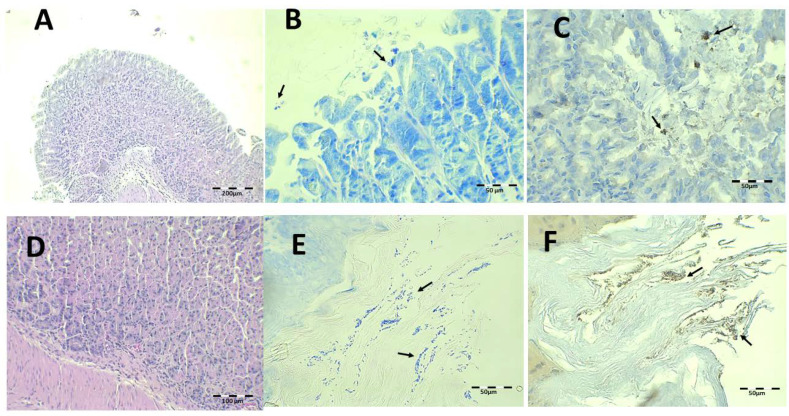
Infected animals treated with 44.7 mg/kg/day of Amox and 22.35 mg/kg/day of Clar in Mucolast^®^ microphotographs of the stomach (**A**–**D**) and esophagus (**E**,**F**), stained with H-E (**A**,**C**), Giemsa (**B**,**E**, arrows indicate *H. pylori* bacteria), and IHC (**C**,**F**, arrows indicate *H. pylori* bacteria).

Finally, histological examination after treatment with Mucolast^®^ at 44.7 and 22.35 mg/kg/day of Amox and Clar, respectively, is shown in Figure 14. There are no histopathological alterations, and there is a minimum amount of inflammatory cell infiltration. However, under Giemsa (Figure 14B,E) and IHC (Figure 14C,F), there is an increased presence of bacteria compared to the equivalent dose of saline solution. Considering the previous results of CFU and the fact that the paste retained bacteria, these bacilli probably lost their activity and were simply retained by the paste.

##### Biochemical Analysis

Several inflammatory markers were evaluated in the gastric mucosa.

**Figure 15:**
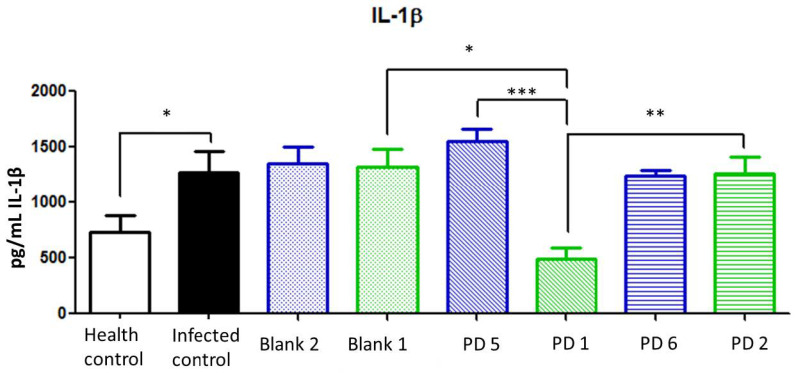
Interleukin (IL)-1β expression (pg/mL) in the stomach after different treatments and controls. Blank 1: Mucolast^®^, Blank 2: PBS. PD1: Amox 89.4 mg/kg/day, Clar 44.7 mg/kg/day in Mucolast^®^. PD2: Amox 44.7 mg/kg/day, Clar 22.35 mg/kg/day in Mucolast^®^. PD5: Amox 89.4 mg/kg/day, Clar 44.7 mg/kg/day in PBS. PD6: Amox 44.7 mg/kg/day, Clar 22.35 mg/kg/day in PBS. * Statistical differences at 0.05, ** statistical differences at 0.01, and *** statistical differences at 0.001.

IL-1β is a potent pro-inflammatory cytokine expressed in *H. pylori* infection, and it is related to tumorigenic-associated gastric cancer [32]. In addition, IL-1β production is associated with parietal cell impairment, which increases achlorhydria and the progression of the disease [2]. According to Figure 15, the IL-1β levels in treatments at 44.7 and 22.35 mg/kg/day of Amox and Clar, respectively, did not show statistical differences with infected nontreated animals. This is also observed in animals treated with 89.4 mg/kg/day of Amox and 44.7 mg/kg/day of Clar in saline solution. On the other hand, animals treated at 89.4 mg/kg/day of Amox and 44.7 mg/kg/day of Clar loaded in the Mucolast^®^ vehicle dramatically reduced the expression of IL-1β compared with non-loaded antibiotics at the same dose (*p* < 0.001) and showed a slight reduction compared with control non-infected animals.

**Figure 16:**
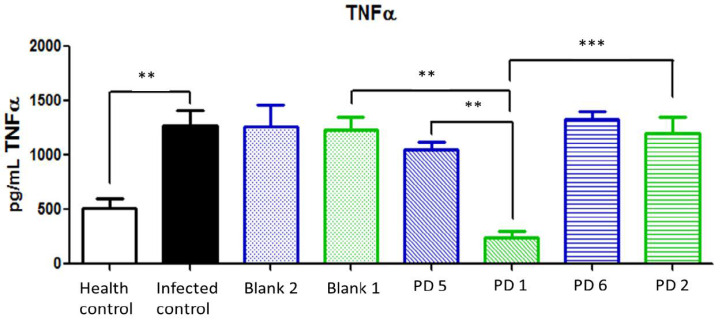
Tumor necrosis factor alpha (TNFα) expression (pg/mL) in the stomach after different treatments and controls. Blank 1: Mucolast^®^, Blank 2: PBS. PD1: Amox 89.4 mg/kg/day, Clar 44.7 mg/kg/day in Mucolast^®^. PD2: Amox 44.7 mg/kg/day, Clar 22.35 mg/kg/day in Mucolast^®^. PD5: Amox 89.4 mg/kg/day, Clar 44.7 mg/kg/day in PBS. PD6: Amox 44.7 mg/kg/day, Clar 22.35 mg/kg/day in PBS. ** Statistical differences at 0.01 and *** statistical differences at 0.001.

TNFα is overexpressed in *H. pylori* infections, and it is related to high levels of infestation and an elevated risk of gastric cancer [2]. Similar to the results for IL-1β, the dose corresponding to 44.7 and 22.35 mg/kg/day doses of Amox and Clar, respectively, loaded in Mucolast^®^ paste had significative differences with the equivalent non-loaded dose and returned TNFα values to the levels found in control healthy animals. A lower dose did not show differences between formulations or with control infected animals (Figure 16).

Prostaglandin E2 (PGE2) is a lipid compound obtained by COX-2 activity. It is involved in the normal function and structure of the gastric mucosa. The overexpression of PGE2 is related to gastric cancer promotion via WNT and/or peroxisome proliferator-activated receptor (PPAR)-δ signaling pathways. In addition, it has been shown that PGE2 has immunosuppressive properties which contribute to cancer progression [33]. Increases in PGE2 levels are secondary to COX-2 overexpression in injured tissue, as one of the rate-limiting factors of its production [34].

**Figure 17:**
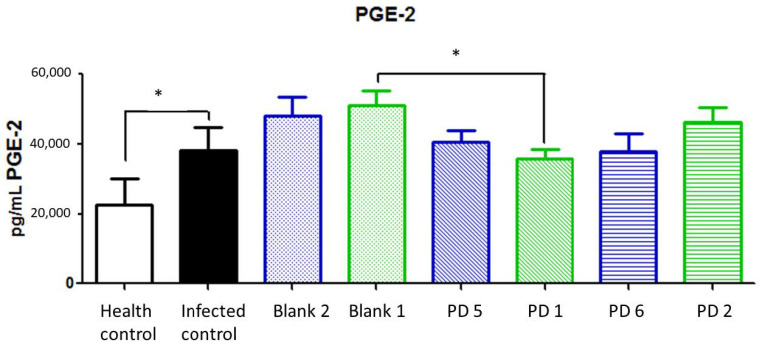
Prostaglandin E2 (PGE2) expression (pg/mL) in stomach after different treatments and controls. Blank 1: Mucolast^®^, Blank 2: PBS. PD1: Amox 89.4 mg/kg/day, Clar 44.7 mg/kg/day in Mucolast^®^. PD2: Amox 44.7 mg/kg/day, Clar 22.35 mg/kg/day in Mucolast^®^. PD5: Amox 89.4 mg/kg/day, Clar 44.7 mg/kg/day in PBS. PD6: Amox 44.7 mg/kg/day, Clar 22.35 mg/kg/day in PBS. * Statistical differences at 0.05.

In this case, no significant differences were observed between treatments, although a reduction trend was observed in 89.4 and 44.7 mg/kg/day doses of Amox and Clar. PGE2 reduction could be a late-stage marker because it is derived from COX-2 expression; thus, the reduction levels could be less than initially expected (Figure 17).

**Figure 18:**
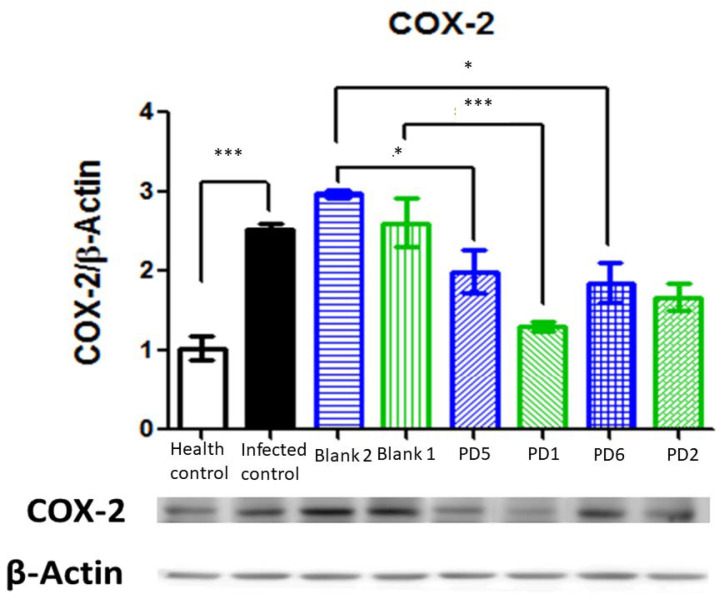
Cyclooxygenase (COX)-2 expression normalized by β-actine in the stomachs after different treatments and controls. Blank 1: Mucolast^®^, Blank 2: PBS. PD1: Amox 89.4 mg/kg/day, Clar 44.7 mg/kg/day in Mucolast^®^. PD2: Amox 44.7 mg/kg/day, Clar 22.35 mg/kg/day in Mucolast^®^. PD5: Amox 89.4 mg/kg/day, Clar 44.7 mg/kg/day in PBS. PD6: Amox 44.7 mg/kg/day, Clar 22.35 mg/kg/day in PBS. * Statistical differences at 0.05 and *** statistical differences at 0.001.

Cyclooxygenase (COX) enzymes include isoform 1, which is expressed constitutively in all tissues, and isoform 2, which is induced after inflammatory stimuli (for example, injury or other cell mediations, such as IL1 and TNFα). COX-2 expression in *H. pylori* gastric lesions seems to be related to the VacA virulence factor [35], and it is also associated with cancer progression [33]. As can be observed in Figure 18, COX-2 is strongly expressed in infected animals and there is a significative reduction in its expression with 89.4 and 44.7 mg/kg/day doses of Amox and Clar. The other treatments, with antibiotics in saline solution and 44.7 and 22.35 mg/kg/day doses of Amox and Clar in Mucolast^®^, also reduced COX-2 expression, although they did not achieve complete eradication.

The histological and biochemical results presented confirm the results described previously regarding the CFU/g found in the stomach. Antibiotic doses corresponding to 89.4 and 44.7 mg/kg/day of Amox and Clar loaded in Mucolast^®^ were superior to the equivalent dose of free antibiotics and exhibited complete eradication of *H. pylori*, as corroborated by the biochemical marker studies and the absence of histological alterations in the stomach. The superiority of the new treatment with the Mucolast^®^ vehicle is assumed to be caused by the topical effect on the stomach, as shown in the pharmacokinetic study due to the high drug concentration at the site of effect.

Further clinical studies will be carried out to evaluate the superior efficacy of antibiotics loaded in Mucolast^®^ compared with standard tablet treatments.

## 4. Conclusions

A new mucoadhesive drug delivery system to administrate Amox and Clar for the treatment of *H. pylori* infections has been studied. Antibiotics are incorporated before administration due to the lack of long-term stability of Amox in the vehicle. Mucolast^®^ was optimized to improve stability and was studied regarding its mucoadhesion and drug release. The modified drug release of both antibiotics was obtained. Pharmacokinetic in vivo studies showed higher drug concentrations in the stomach compared to free antibiotics at equivalent doses while maintaining approximately the same systemic drug exposure. The local effect of the higher antibiotic concentration at the site of action is probably the reason for the increased efficacy of the product in terms of the eradication of *H. pylori* infection in mice. The histopathological and biochemical biomarkers confirmed the eradication results at doses equivalent to 89.4 and 44.7 mg/kg/day of Amox and Clar loaded in Mucolast^®^. This is a promising option to improve the therapeutic treatment of stomach ulcers caused by *H. pylori.*

## 5. Patents

WO2017162822 compositions for mucosal adhesion and uses thereof.

## Figures and Tables

**Table 1:** Pharmacokinetic (PK) groups per formulation, time, and dose. Amox: amoxicillin; Clar: clarithromycin.

Group	Vehicle	Amox	Clar	Subgroup	Sacrifice Time (h)
PK 1	Mucolast^®^	89.4 mg/kg/day	44.7 mg/kg/day	a	0
				b	1
				c	2
				d	4
				e	6
				f	24
PK 2	Mucolast^®^	44.7 mg/kg/day	22.35 mg/kg/day	a	0
				b	1
				c	2
				d	4
				e	6
				f	24
PK 3	Mucolast^®^	8.94 mg/kg/day	4.47 mg/kg/day	a	0
				b	1
				c	2
				d	4
				e	6
				f	24
PK 4	PBS	89.4 mg/kg/day	44.7 mg/kg/day	a	0
				b	1
				c	2
				d	4
				e	6
				f	24
PK 5	PBS	44.7 mg/kg/day	22.35 mg/kg/day	a	0
				b	1
				c	2
				d	4
				e	6
				f	24
PK 6	PBS	8.94 mg/kg/day	4.47 mg/kg/day	a	0
				b	1
				c	2
				d	4
				e	6
				f	24
Blank animals				--	--

**Table 2:** Efficacy of groups per formulation, time, and dose.

Treatment	Vehicle	Amox Dose	Clar Dose	n
PD 1	Mucolast^®^	89.4 mg/kg/day	44.7 mg/kg/day	11
PD 2	Mucolast^®^	44.7 mg/kg/day	22.35 mg/kg/day	12
PD 3	Mucolast^®^	8.94 mg/kg/day	4.47 mg/kg/day	8
PD 4	PBS	357.5 mg/kg/day	178.8 mg/kg/day	4
PD 5	PBS	89.4 mg/kg/day	44.7 mg/kg/day	8
PD 6	PBS	44.7 mg/kg/day	22.35 mg/kg/day	8
Blank 1	Mucolast^®^	-	-	12
Blank 2	PBS	-	-	10

**Table 3:** Results of the rheological behavior, mucoadhesion time, and floatability.

Formula	Xanthan Gum (%)	Xanthan Gum Hydration Time (h)	Xanthan Gum Hydration Temp (°C)	Viscosity (mPA·s)	G′ Slope	R^2^	G′ (0.1 HZ)	Δ	Mucoadhe. Time (min)	Floatability
1	2.00	2	70	15,300	0.2836	0.944	8.83	1.25	400	NO
2	2.00	2	75	12,500	0.2037	0.852	6.33	1.41	NP	NO
3	3.00	2.5	70	45,825	0.2201	0.971	76.20	0.63	238	NO
4	2.50	2	70	11,475	0.2183	0.924	14.57	0.93	400	NO

NP: not performed (the R^2^ parameter was the worst obtained among formulations and was not considered suitable).

**Table 4:** Model fit and release parameters of Amox and Clar (high and low doses). AIC: Akaike Information Criterion. Bold indicates the model selected based on the lowest AIC. β is a dimensionless parameter. High dose: 89.4 mg/kg/day of Amox and 44.7 mg/kg/day of Clar. Low dose: 8.94 mg/kg/day of Amox and 4.47 mg/kg/day of Clar.

Drug	Model	AIC Value	Parameters	Value (Mean ± SD)
Amox Low Dose	First-order	29.0136	K (h^−1^)	0.291 ± 0.091
	Higuchi	28.3848	K_h_ (% h^−1/2^)	23.862 ± 7.658
	Weibull	15.2055	β	0.610 ± 0.050
			t_d_ (h)	6.063 ± 0.921
	**Kosmeyer–Peppas**	**13.2034**	**K_k-p_ (% h^−n^)**	**20.572 ± 1.120**
			**n**	**0.610 ± 0.043**
Amox High Dose	First-order	25.8038	K (h^−1^)	0.180 ± 0.064
	Higuchi	34.5868	K_h_ (% h^−1/2^)	20.576 ± 8.997
	Weibull	15.6268	β	0.716 ± 0.044
			t_d_ (h)	6.223 ± 0.769
	**Kosmeyer–Peppas**	**13.6268**	**K_k-p_ (% h^−n^)**	**15.324 ± 1.232**
			**n**	**0.717 ± 0.031**
Clar Low Dose	First-order	30.4070	K (h^−1^)	0.376 ± 0.062
	**Higuchi**	**25.7367**	**K_h_ (% h^−1/2^)**	**14.612 ± 2.021**
	Weibull	29.0195	β	0.548 ± 0.099
			t_d_ (h)	3.101 ± 1.757
	Kosmeyer–Peppas	27.0192	K_k-p_ (% h^−n^)	13.563 ± 2.884
			n	0.547 ± 0.091
Clar High Dose	First-order	33.9579	K (h^−1^)	0.419 ± 0.089
	**Higuchi**	**24.7305**	**K_h_ (% h^−1/2^)**	**15.887 ± 1.82**
	Weibull	28.7287	β	0.502 ± 0.090
			t_d_ (h)	5.776 ± 1.897
	Kosmeyer–Peppas	26.7287	K_k-p_ (% h^−n^)	15.843 ± 2.061
			n	0.502 ± 0.100

**Table 5:** Amox and Clar area under the curve (AUC) plasma values after the administration of antibiotics in Mucolast^®^ or saline solution at three different doses.

Group	Tissue	Vehicle	Dose Amox	AUC_all_ Amox (µg mL^−1^ h^−1^)	Dose Clar	AUC_all_ Clar (ng mL^−1^ h^−1^)
**PK 1**	Plasma	Mucolast^®^	89.4 mg/kg/day	75.9	44.7 mg/kg/day	**5470.5**
**PK 2**		Mucolast^®^	44.7 mg/kg/day	63.7	22.35 mg/kg/day	**3371.5**
**PK 3**		Mucolast^®^	8.94 mg/kg/day	10.7	4.47 mg/kg/day	**1083.6**
**PK 4**		PBS	89.4 mg/kg/day	75.1	44.7 mg/kg/day	**5426.0**
**PK 5**		PBS	44.7 mg/kg/day	26.6	22.35 mg/kg/day	**2326.1**
**PK 6**		**PBS**	**8.94 mg/kg/day**	**10.2**	**4.47 mg/kg/day**	**672.1**

**Table 6:** Amox and Clar AUC mucosa values after the administration of antibiotics in Mucolast^®^ or saline solution at three different doses.

Group	Tissue	Vehicle	Dose Amox	AUC_all_ Amox (µg g^−1^ h^−1^)	Dose Clar	AUC_all_ Clar (µg g^−1^ h^−1^)
PK 1	Gastric mucosa	Mucolast^®^	89.4 mg/kg/day	10,553.9	44.7 mg/kg/day	3035.7
PK 2		Mucolast^®^	44.7 mg/kg/day	13,911.2	22.35 mg/kg/day	4158.7
PK 3		Mucolast^®^	8.94 mg/kg/day	1079.3	4.47 mg/kg/day	576.4
PK 4		PBS	89.4 mg/kg/day	206.4	44.7 mg/kg/day	94.0
PK 5		PBS	44.7 mg/kg/day	78.6	22.35 mg/kg/day	27.7
PK 6		PBS	8.94 mg/kg/day	31.8	4.47 mg/kg/day	19.4

**Table 7:** Amox and Clar AUC stomach values after the administration of antibiotics in Mucolast^®^ or saline solution at three different doses.

Group	Tissue	Vehicle	Dose Amox	AUC_all_ Amox (µg g^−1^ h^−1^)	Dose Clar	AUC_all_ Clar (µg g^−1^ h^−1^)
PK 1	Stomach	Mucolast^®^	89.4 mg/kg/day	1438.2	44.7 mg/kg/day	169.0
PK 2		Mucolast^®^	44.7 mg/kg/day	1639.1	22.35 mg/kg/day	557.5
PK 3		Mucolast^®^	8.94 mg/kg/day	61.5	4.47 mg/kg/day	84.1
PK 4		PBS	89.4 mg/kg/day	68.4	44.7 mg/kg/day	34.6
PK 5		PBS	44.7 mg/kg/day	30.4	22.35 mg/kg/day	14.1
PK 6		PBS	8.94 mg/kg/day	10.3	4.47 mg/kg/day	3.6

**Table 8:** *H. pylori* bacterial count (Colony Forming Units/mL (CFU/g)) in mice stomachs at 14 and 21 days after inoculation and in healthy controls.

Group	n	CFU/g (mean ± SD)
Heath control	5	0.00 ± 0.00
*H. pylori* 2 weeks	5	6.15 ± 0.84
*H. pylori* 3 weeks	5	6.54 ± 1.10

## Data Availability

The data presented in this study are available on request from the corresponding author. The data are not publicly available due to intellectual properties restrictions.
